# Introduction of Systematized Nomenclature of Medicine–Clinical Terms Coding Into an Electronic Health Record and Evaluation of its Impact: Qualitative and Quantitative Study

**DOI:** 10.2196/29532

**Published:** 2021-11-23

**Authors:** Tanya Pankhurst, Felicity Evison, Jolene Atia, Suzy Gallier, Jamie Coleman, Simon Ball, Deborah McKee, Steven Ryan, Ruth Black

**Affiliations:** 1 NHS Foundation Trust University Hospitals Birmingham Birmingham United Kingdom; 2 Health Data Research UK (HDR-UK) University of Birmingham Birmingham United Kingdom; 3 Institute for Global Health Innovation (IGHI) Imperial College London London United Kingdom

**Keywords:** coding standards, clinical decision support, Clinician led design, clinician reported experience, clinical usability, data sharing, diagnoses, electronic health records, electronic health record standards, health data exchange, health data research, International Classification of Diseases version 10 (ICD-10), National Health Service Blueprint, patient diagnoses, population health, problem list, research, Systematized Nomenclature Of Medicine–Clinical Terms (SNOMED-CT), use of electronic health data, user-led design

## Abstract

**Background:**

This study describes the conversion within an existing electronic health record (EHR) from the *International Classification of Diseases, Tenth Revision* coding system to the SNOMED-CT (Systematized Nomenclature of Medicine–Clinical Terms) for the collection of patient histories and diagnoses. The setting is a large acute hospital that is designing and building its own EHR. Well-designed EHRs create opportunities for continuous data collection, which can be used in clinical decision support rules to drive patient safety. Collected data can be exchanged across health care systems to support patients in all health care settings. Data can be used for research to prevent diseases and protect future populations.

**Objective:**

The aim of this study was to migrate a current EHR, with all relevant patient data, to the SNOMED-CT coding system to optimize clinical use and clinical decision support, facilitate data sharing across organizational boundaries for national programs, and enable remodeling of medical pathways.

**Methods:**

The study used qualitative and quantitative data to understand the successes and gaps in the project, clinician attitudes toward the new tool, and the future use of the tool.

**Results:**

The new coding system (*tool*) was well received and immediately widely used in all specialties. This resulted in increased, accurate, and clinically relevant data collection. Clinicians appreciated the increased depth and detail of the new coding, welcomed the potential for both data sharing and research, and provided extensive feedback for further development.

**Conclusions:**

Successful implementation of the new system aligned the University Hospitals Birmingham NHS Foundation Trust with national strategy and can be used as a blueprint for similar projects in other health care settings.

## Introduction

### Background

The Digital Age has brought fast and convenient technology to almost all industries, and latterly to health care. The current system in Britain is divided between primary health care, where general practitioners increasingly manage chronic disease, and secondary and tertiary care, which largely remain modeled on hospitals. General practitioner records have been electronic for 20 years [1], but hospital records are only being ubiquitously considered for digital conversion now. These electronic health records (EHRs) are variable and often either overly simplistic [2] or require so much data input that they overburden clinicians [3]. EHRs have grown up separately and most still do not allow easy sharing of data, a situation further compounded by variability of the data themselves and lack of standardized coding [4]. Paucity of data exchange is not only a barrier to treating patients [5] but also prevents remodeling of medical pathways [6] locking the National Health Service (NHS) into artificial divisions between primary and secondary care. NHS Digital has issued guidance on the use of standards, including the universal use of the SNOMED-CT (Systematized Nomenclature of Medicine–Clinical Terms) for diagnoses [7] and other standards for medications, data messaging, and patient identification [8].

University Hospitals Birmingham (UHB) is a large secondary and tertiary referral NHS Foundation Trust comprising 4 hospitals and community services across Birmingham, the second largest city in the United Kingdom. UHB has an annual turnover of £1.4 (US $1.88) billion and treats 2.2 million people per year [9]. Across its 4 sites it has 3000 inpatient beds and employs more than 20,000 people. UHB has been at the forefront of digital innovation and is unique in the United Kingdom in employing programmers to build its own EHR. The organization was recognized as a global digital exemplar in 2017 [10]. It has strong links to primary care and is engaged in building regional data-sharing platforms [11]. Therefore, it is highly influential in digital health care development regionally and nationally in the United Kingdom.

The University Hospitals Birmingham NHS Foundation Trust’s (the Trust, hereafter) central EHR, named *Prescribing Information and Communication System* (*PICS*), [12] comprises contemporaneous clinical noting; electronic prescribing and medicines administration; electronic observations; electronic ordering; patient barcoding; and electronic results display and complex clinical decision support (CDS). UHB builds other software, including clinical and patient portals.

Electronic health care systems are ubiquitously used, and their design has been controlled by clinicians over many years. Diagnostic coding within PICS was based on the *International Classification of Diseases, Tenth Revision* (*ICD-10*) [13], which is the current system used for payment of hospitals. The *ICD-10* was originally designed for population studies and is, therefore, not ideally suited to hospital settings. Clinicians often cannot find diagnoses they need because detailed or rare conditions are listed under *any other...*. Consequently, diagnoses are often not recorded and are therefore lost to use within any CDS rules and lost to data exchange beyond the organizational boundaries.

Implementation of SNOMED-CT coding into PICS was predicted to allow clinicians to more accurately enter detailed diagnoses, enable more complex CDS, and facilitate more accurate data collection, provided the tool was intuitive. In addition, there was a strong interest from the central government and the public to share health data. Primary care has already encoded diagnoses using SNOMED-CT, and similar data collection in hospitals would enable data exchange across previously incompatible data entry applications. This approach enables the remodeling of medical pathways and begins to address the siloed nature of medical care.

### Objectives

The organization’s mission is to improve the health of patients and communities, and UHB is committed to transformation through consistent innovation across digital systems. The goals of UHB align with national expectations around standardized electronic data collection and the easy and seamless sharing of data [14]. In addition, clinicians require a more enriched and relevant system to record medical diagnoses. To address these goals, this study sought to establish coding within all components of the EHR based on the SNOMED-CT by April 2020. Therefore, the question was whether the current EHR, with all relevant patient data, could be migrated to the SNOMED-CT coding system, to optimize clinical use and CDS and facilitate data sharing across organizational boundaries for national programs and remodeling of medical pathways.

## Methods

### Overview

The formal methodology was designed to assess the success of the tool (utility or clinical relevance), success of mapping and safeguarding CDS rules in the software, and successful data collection for direct clinical care, research, and data exchange. This used pre-existing data sets, interviews, focus groups, and surveys of the stakeholders, and formal assessment of the use of the tool with quantitative data, using a mixed methods approach.

### Existing Data Sets: Fixed Points

The design and build of the SNOMED-CT tool were created by clinicians and developers using an iterative model. Design meetings included clinicians, developers, business analysts, and a project manager, and a specification document was initially developed; development followed, and demonstrations of the software build and modifications were reviewed as they progressed over time. Many of the questions that the study explores in the postlaunch analysis (eg, “Is the tool useful?”) can be analyzed in this predata set, where the clinicians and developers worked together (eg, to create a tool that they believed *would* have clinical utility). The project specification document, specifically the user stories, provides a fixed point for analysis (Multimedia Appendix 1).

### Surveys

A survey targeted at a large sample of clinicians after the launch of the SNOMED-CT tool assessed clinician opinion on utility and improvements in clinical care associated with the tool. The survey was sent by email across the Trust via Typeform [15] to all junior doctors and all consultants on the master staff index. Senior nurses (the only nursing group using the tool) were sent the same survey via the divisional leads; Allied Health Professionals (AHPs) were sampled using group leaders.

The SNOMED-CT tool was designed to collect consistent and standardized data, independent of which health care workers from the multidisciplinary team were collecting it. Thus, the tool had to work for doctors, nurses, and AHPs and be intuitive and accessible to junior and senior staff. To qualitatively assess whether this was successful, the study used a wide sample of users.

The sampling strategy was typical purposeful sampling, aiming to represent the average clinical situation and sample across all types of staff using the new tool. The sampling aimed to highlight the experience of this tool and understand the typical, normal, and average experience of a clinician using the tool [16].

Survey questions were developed with a focus on understanding staff experiences and future expectations regarding the use of a tool in constructing a problem list within a patient record. The tool’s utility in gathering data for direct clinical care, onward sharing, and the creation of research sets was explored. There were 7 elements to the survey (Multimedia Appendix 2) designed on a Likert scale [17] with one open-ended question, allowing for more detailed data collection.

From previous surveys of this type within the organization, the expected response rate was about 20%. The survey was designed for easy and rapid completion. Clinicians are busy, and traditionally ignore feedback requests. On the basis of previous experience and supported by market research [18], it was known that surveys exceeding 5 minutes would not be completed. The survey was therefore much shorter than a classical qualitative study survey, but accrued as much qualitative information as possible.

The validity and reliability of these items were tested by adequate engagement to saturation (the survey was repeatedly sent out to the staff lists until there were no new elements to answers) [19] and by triangulation or crystallization [20] of pre-existing data sets, focus groups, and data collection.

### Interviews

Senior programmer interviews created an understanding of build success in relation to the original specification. Senior developers worked closely with clinicians and identified flaws in the design and implementation. Interviews with senior developers revealed difficulties and workarounds in the course of the build and illuminated reasons for subsequent clinician behavior. Interviews were designed as one-time sessions with each of the 2 principal developers in a meeting room in the building in which they worked. The interviews were informal and semistructured, asked open questions and allowed for follow-up and probing. The interviews were recorded and transcribed later, and notes were taken during the interviews. The questions (Multimedia Appendix 3) were designed to investigate, from the developers’ perspective, the success of the project, and the aspects that they viewed as strengths and weaknesses. Interviews also queried how developers viewed users and requested opinions about the implications of the project beyond the software build. The questions were deliberately open [16].

In similar previous projects, it was noted that developers who worked closely with clinicians often had insight into the software and possible enhancements that would improve user experience and data exchange. Developers are often reserved in their judgment and often do not proffer an opinion unless specifically asked.

### Focus Groups and Email Feedback

Focus groups aimed to understand the validity and utility of the final product from clinicians involved in the iterative design and build of the software. In addition, unsolicited feedback from clinicians is a frequent part of the EHR build, and email feedback was therefore included in the thematic analysis. There were up to 50 clinicians involved in the design of the EHR, but the focus groups included between 5 and 10 participants, as this was an optimal number to support vibrant discussion while allowing all voices to be heard [21], thus using nonprobability unique sampling [19]. Clinicians who were invited were the ones most actively involved in the design and build, and to whom the tool was most relevant (because of direct clinical care, a research interest, or an interest in onward data sharing). Additional email feedback was not requested, but extensive feedback from this source was provided and was therefore included in the analysis. Clinicians in the focus groups and those who provided email feedback were likely to be a subset of those responding to the survey.

**Figure 1:**
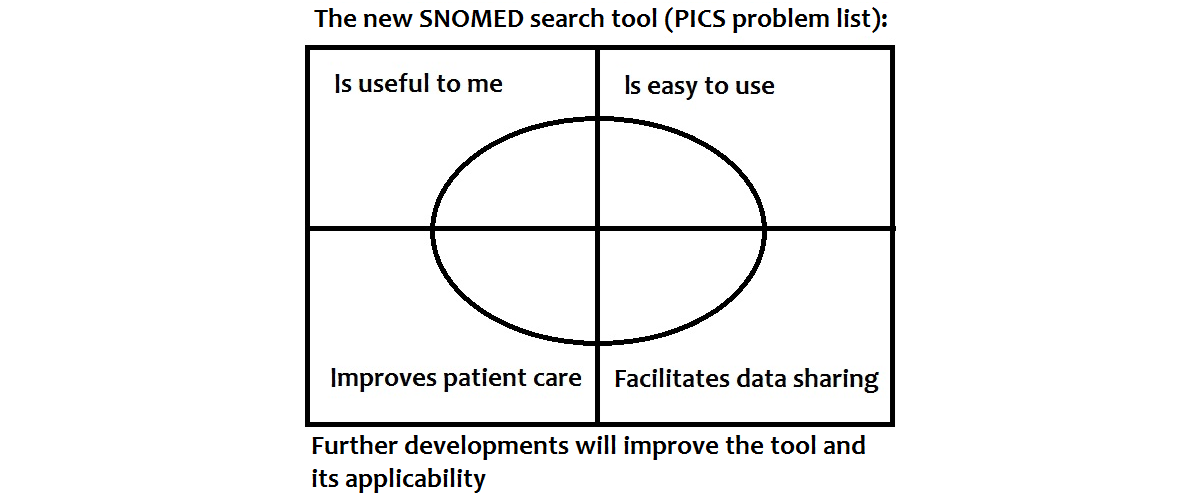
Importance of the themes to the users. PICS: Prescribing Information and Communication System; SNOMED-CT: Systematized Nomenclature of Medicine–Clinical Terms.

Two focus groups were conducted with 6 and 8 clinicians. The focus groups were informal and semistructured with prompts (Figure 1), which not only allowed for expression of opinion on the entire conceptual framework but also allowed for discussion, so that aspects of the project that had not been previously understood by the investigators were revealed.

The questions were designed to understand the utility of the tool in terms of clinical use and acceptability. This requires careful assessment, as clinicians may welcome even clunky digital tools if they think that patient care is improved. In relation to the conceptual framework, questions on the applicability of the tool to direct clinical care, research, and data sharing were also explored.

### Data Analysis

#### Pre-existing Data Sets or Fixed Points

These predated the study and were analyzed by extracting data against the key themes of improving care, improving access to data, and ensuring usability of the tool. The data set comprised user stories from a specification document developed iteratively with developers and clinicians. Suggestions and actions incorporated during the data build were analyzed and used to understand whether the same points were discussed in the survey responses and focus groups (eg, were they addressed in the build? If they were not, why not?). The documents were coded in a similar manner to that of the focus groups, which allowed theming and issue spotting.

#### Surveys

Descriptive statistical analyses were conducted on the results after all the surveys were submitted. For all questions answered on a Likert scale, responses to each question were analyzed as whole numbers in each group. Binary questions were presented as percentages. The analysis of open questions gathered themes with the quoted examples.

#### Interviews, Focus Groups, and Email Feedback

Interviews and focus groups were recorded and transcribed, and thoughts, concerns, and conclusions documented by the investigator during or immediately after the interviews or focus groups. The transcription was coded and scored on a matrix (Figure 1) for frequency, concern, and time spent discussing each issue and where they overlapped using a multifactorial balancing test-like methodology [22]. Themes or issues were identified and cross-referenced with survey results and pre-existing data sets.

Informal email feedback was collected by the chief investigator and incorporated into the thematic analysis.

#### Use Data

Data were collected digitally on the use of the SNOMED-CT tool across the entire organization by the Informatics Department. The following data were collected: total number of users, by area and specialty; number of terms used, by user and by specialty; and application over time (for the first 4 months of use) to understand whether the use was increasing or waning. These data were presented graphically as absolute numbers and as the percentage use of the total user base.

### Ethics

Human subjects were involved in this research, and participation was voluntary and confidential. Informed consent was obtained from all participants, including for recording and storing of information, and consent could be withdrawn at any time until results were collated in an anonymized manner. Any quotations from the interviews and focus groups were only used with consent. It is unlikely in a study of this type that interviews or focus groups would cause ethical issues to be raised as private behavior was not the subject of this study [16]. The data were stored securely and confidentially.

Bias arising from the dual role of the researcher as a designer or user and evaluator of the tool [16] was mitigated by processes already in place. All software build within the organization was controlled by a project group, with a project manager, business analyst, standard testing, and rollout procedures. Although researchers have invested in the project, many software developments to date have required extensive iterations to ensure utility, and this was expected to be necessary in this project also.

## Results

### Overview

Data collection took place during September 2019-December 2019 and was conducted at UHB, United Kingdom. Statistics for the problem list in PICS were gathered by the Informatics Department, from the date of the tool release into PICS software, September 2019, to the end of December 2019.

The 5 key themes with subthemes that emerged from this study are detailed in Table 1.

**Table 1:** Themes emerging from user feedback, with positive and negative aspects.

Theme and subtheme		Positive feedback	Negative feedback
**Design commitment and project success**			
	In general	All project objectives met Positive user feedback	—^a^
	Usability	High use Easy to find Search tool affective	Changes to user interface needed Some terms not found Too many choices
	Engagement	Things clear and in the same place	Time constraints Incentive low
Data quality		Much more detailed data Enriched data sets	Incomplete data Too much data
Improving patient care		Use in safety rules Improves communication	Lists too long Lists not sorted in my order
Research		Data rich source	Too many codes
**Data** **sharing**			
	In general	Standardized data collection Sharing with general practitioners Sharing with other hospitals	—
	Standards and payment	Provides standard	—

^a^No data obtained.

Questionnaires were sent to 585 junior doctors, 315 consultants, 40 senior nurses, and 20 AHPs. In total, 15.3% (147/960) responded to the survey. The survey was anonymous; thus, the role of the respondents was not recorded.

### Design, Commitment, and Project Success

The project delivered the planned tool. The requirement for programmer time was high, up to 40 hours per week in the last month of the project, with extensive preceding groundwork for the project team. A total of 300,000 new codes were added to the EHR, and 15,000 old codes were mapped to new codes. Because of the considerably increased detail in SNOMED-CT compared with the *ICD-10*, this mapping was complicated and multiple SNOMED-CT codes had to be mapped back to each *ICD-10* code. In addition to coding, the existing EHR had over 1000 existing CDS rules, all of which were successfully mapped to new codes. All these changes required a new user interface (UI), which was iteratively built with clinicians.

The programmers who built the new tool felt that the overall project was well organized and well managed. However, programmers were frustrated at the time required for mapping and checking the codes. The programmers felt that coders should have been involved earlier and more extensively. Before the project, the programmers were concerned that loading thousands of new codes would slow down the application, reducing both the speed of the search and the EHR application itself. This concern did not materialize because search speeds and EHR stability were not impacted by the loading of a large number of new codes.

Programmers further commented that predicting problems in organizing and anticipating questions was difficult in innovation projects of this scope. Fixed data sources (Multimedia Appendix 1) included specification documents and these outlined *user stories* identifying what users articulated as their need. All phase 1 requirements were delivered. Some modifications were requested in the feedback, which are outlined in the themes below. Similar to the programmers, users fed back that the project was successful. Users had assumed that it would be successful, as the build of EHR in our organization has a good track record for the delivery of successful tools.

### Usability

**Figure 2:**
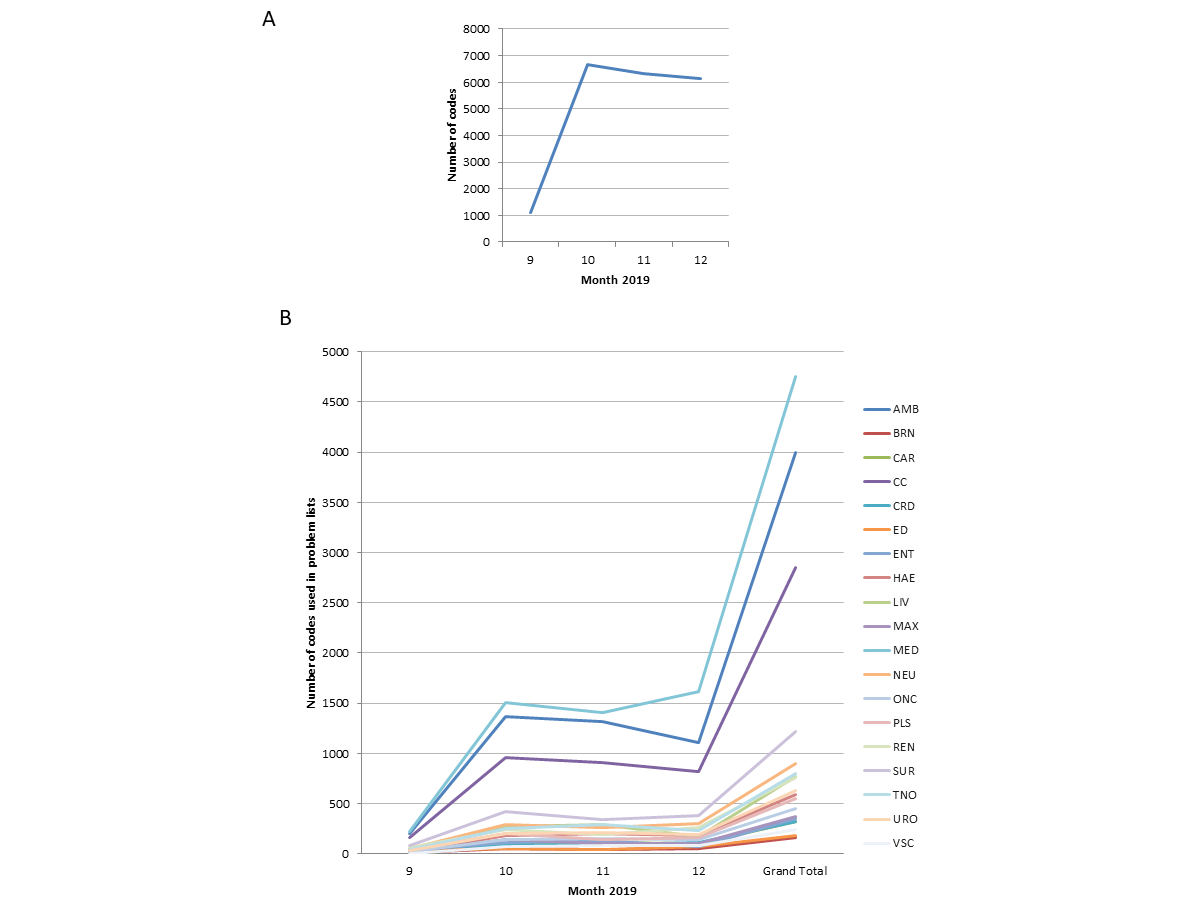
Use of the new tool. (A) Monthly problem list use. (B) Use of problem lists by clinician type. AMB: ambulatory care; BRN: Burns Medicine; CAR: cardiac surgery; CC: critical care; CRD: cardiology; ED: emergency medicine; ENT: ear nose and throat; HAE: haematology; LIV: liver medicine; MAX: maxillo-facial surgery; MED: general medicine; NEU: neurology; ONC: oncology; PLS: plastic surgery; REN: renal medicine; SUR: general surgery; TNO: trauma and orthopaedics; URO: urology; VSC: vascular surgery.

This tool has been widely used. After the first month, codes entered into the EHR rapidly rose to between 6000 and 7000 a month and continued to be high over the 4 months of the study (Figure 2). This was higher than expected, as no specific communications or *advertising* was put out to the organization. The expectation is that the number of codes entered each month will fall as more returning patients have most of their medical history added to the EHR. It is anticipated that a steady state will be reached in approximately 12 months with only new patients or new problems being added to the system.

Codes were used in all specialties. As expected, the highest use was in general medicine, short-stay ambulatory care, and critical care, but use was seen across all hospital specialties (Figure 2). Most users were doctors and a significant minority nurses. Small numbers of pharmacists and AHPs were starting to use the tool during the study period, and this is expected to rise as these groups of professionals generally follow doctors’ behavior.

**Figure 3:**
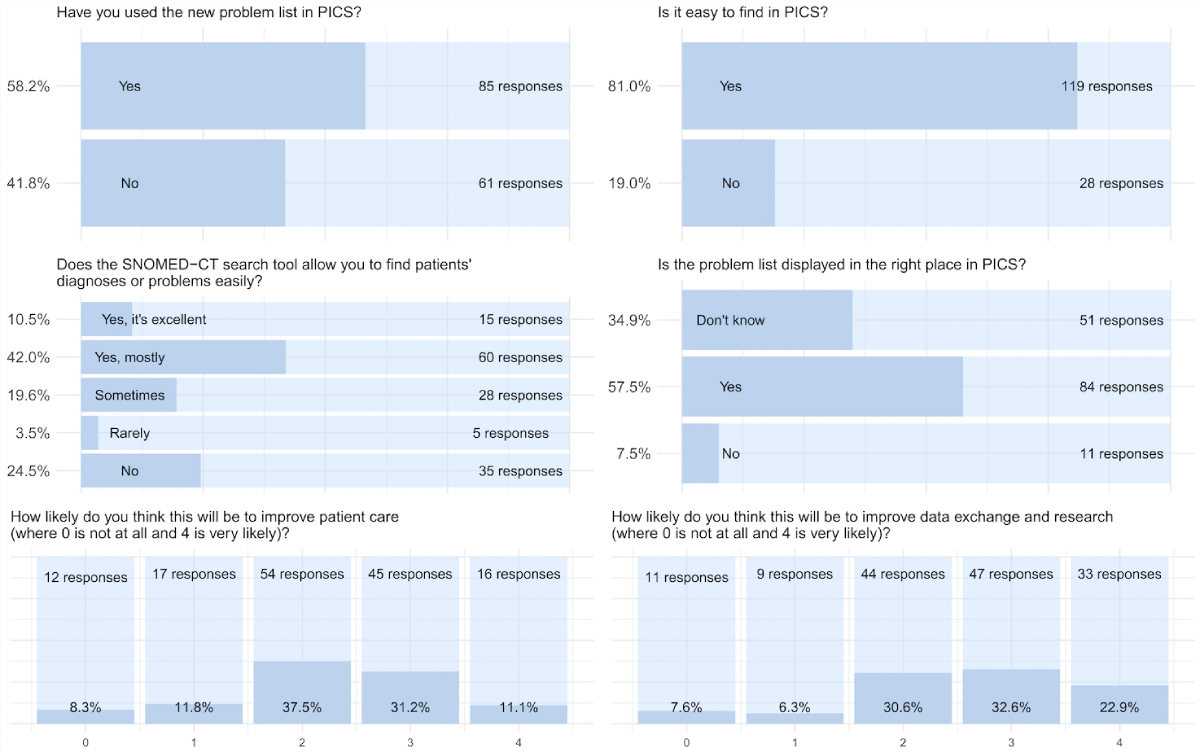
Questionnaire summary for user feedback. PICS: Prescribing Information and Communication System; SNOMED-CT: Systematized Nomenclature of Medicine–Clinical Terms.

Most study participants thought the tool was easy to find (Figure 3) and that diagnoses could be searched for effectively. Most participants also thought that the new problem list was displayed in the right places in the EHR. There were several examples of user feedback requesting changes to the UI; dates did not appear until final commitment to the record that confused some users, and users suggested the addition of abbreviations in the search box.

Most of the required diagnoses seemed to be available, although ophthalmology was reported to be sparse, and some specific diagnoses were not found. Some doctors asked how to add diagnoses to the SNOMED-CT itself. Overall, however, users more frequently commented that there were too many diagnoses rather than too few, and they did not understand that synonyms mapped back to the same codes (Textbox 1).

More fundamentally, surgeons commented that *end* dates did not make sense for operations. They felt that even though the *problem* had ended, surgical history should persist on diagnostic lists. Specific dates for some diagnoses were also thought to be irrelevant. Textbox 1 below is a compilation of free text data from the combined methodology, detailing respondents’ perspectives around the core areas of utility, diagnosis search, and dating diagnoses.

**Usability of new tool.**

**The tool was easy to use**

- “More abbreviations would make search engine more efficient” [user]
- “Agree it’s unlikely that we will find anything better for our speciality” [consultant in cardiac surgery]

**Users could find the right diagnoses but some additions were needed**

- “Even a small number of extra terms for the VAD [ventricular assist device] complication would be very useful and of course there are new devices coming out that aren't covered” [doctor]
- “I’ve tried quite a few orthopaedic trauma diagnoses and it seems comprehensive” [orthopedic surgeon]
- “The search option for some of the diagnoses may need improvement. For example, when I type TB, several tuberculosis- related diagnoses are available. Miliary TB however only appears if I type in Miliary. Similarly some diagnoses seem to have been only tagged to human immunodeficiency virus rather than HIV (eg, Lymphoma with HIV)” [HIV consultant]
- “Only one condition (glaucoma) in ophthalmology section” [ophthalmology doctor]
- “There are still some diagnoses missing from the list, for example Non-specific interstitial pneumonia. Is there any facility for us to add diagnoses to the list?” [HIV consultant]

**Large numbers of diagnoses to choose from**

- “So many to choose from when updating problem list” [doctor]
- “Multiple names for the same thing an issue” [anesthetic consultant]
- “It is quite time consuming to enter data and sometimes it is hard to find the exact diagnosis needed” [user]
- “Many of the diagnoses are repeated so it is not clear which to put down. This may make auditing tricky” [user]
- “Agree. Better than ICD10. There are often multiple options for the same disease -are they automatically clustered together for analysis or would you need to do this manually? CT concept seems to be mainly irrelevant stuff” [hematology consultant]

**Modifications of dates needed**

- “One of the slight frustrations is that when you enter a procedure, for example repair of extensor tendon of hand, the start date has to be different from the end date” [hand surgeon]
- “Some diagnosis patients tend to remember the date like MI [myocardial infarction]. Others such as hypertension are hard to date exactly” [doctor]
- “It seems a little clunky; perhaps I am using it wrong. When I try to enter comorbidities, the SNOMED-CT diagnosis disappears I try to put in the start date” [user]
- “I commonly find a correct problem with search and when I click it just disappears rather than gets added. I am not sure what I am doing wrong but the interface may need attention” [user]

### Engagement

The issue of clinicians not entering all relevant diagnoses was raised many times*,* but as these are persistent data, users thought these may accumulate over time*.* Doctors felt motivated to enter data but were restricted by time constraints and demotivated by the perception that other doctors and other staff members were not doing a share of the work*.* Nurses, patients, and coders were all expected to be potentially powerful contributors to the problem list. Finally, education was raised as important in user engagement.

### Data Quality

Users expressed that the data needed to be correct and updated. Ensuring that all users entered correct and complete lists was thought to be potentially problematic, and deletion of irrelevant or incorrect items from the lists was raised as a concern. Typical feedback about the quality of data entry and barriers to the affective gathering of data are illustrated in Textbox 2.

**Data entry and data quality.**

**Data entry improved by the new tool**

- “Once all the data was in there updating it wouldn’t take too long” [hand surgeon]
- “In clinic now I feel inclined to do things properly” [oncologist]

**Barriers to data entry**

- “Ideally I should do this with all my patients but there is simply not enough time (or perhaps incentive) to do this for all the OPD [outpatient department] patients I see. I do this for my day case patients currently” [user]
- “Too much effort needs to go into entering data, a huge amount of time went into this in a previous Trust but responsibility and effort decrease over time and I’m worried that this may happen here” [nephrologist]
- “Used it today and the PPM [permenant pacemaker] hadn't been entered by the team...still relies on the entry! I hope that my entering the PPM will stay on prescribing information and communication system for all future episodes” [user]
- “The challenge will be making sure that people use it so that it becomes habit” [user]
- “I think the main challenge will be to get enough doctors, of all grades and specialties, to use it to populate the list accurately. The second challenge is how we will know.”
- “I tend to put some stuff in. Not everyone is doing this. In radiology they are not adding” [vascular surgeon]

**Quality of data entry**

- “If I ask a junior doctor to add problems in the problem list they will miss lots of stuff. How can we ensure quality” [ICU consultant]
- “I think we need to take the history properly” [vascular surgeon]
- “We need to make sure things are deleted as well as added” [neurologist]
- “It will only improve things if the user is taken to it and encouraged to review and increase content” [user]
- “Data accuracy is an issue – if I’m coding other people’s diagnoses I’d probably get this wrong” [doctor]

**Involvement of patients and the multidisciplinary team**

- “If engage with it will be useful, but often find there is only half the patients’ past medical history is there. Is there scope that this could be update by pharmacists so when they are taking meds rec they ensure that it is updated on the system as well” [user]
- “The main problem I see with this is like before whether doctors are inputting ‘problem list’. I know that when patients come to [the] angio[graphy suite] it is unlikely that any one will add anything. Can the coders do something with this??” [user]
- “Nurses are good at taking a history – they should put this in there” [ICU consultant]
- “Why don’t we give the patients a copy and ask them ‘is this accurate’?” [upper gastrointestinal surgeon]

### Improving Patient Care

In the questionnaire, users were ambivalent about the tool positively impacting patient care (Figure 3). However, in focus groups, clinicians thought that the problem list positively contributed to patient care and could have wide implications for safety rules. It was also recognized as improving communication*.*

There were concerns about lists becoming too long and that loss of human sorting of lists could lead to a loss of nuance. Many doctors use clinical letters to present a list of patient problems, which are subtly changed in each specialty with a different ordering or grouping of problems. Doctors may also exclude problems that are less relevant to their specialty or add details that are not understood (or transcribed) by other specialists. Building relevant patient lists that cater for all is probably the most challenging aspect of building relevant electronic lists. The current situation in which some information is missed or excluded by doctors may be dangerous, but an alternative that overloads clinicians with information is also not ideal. Pragmatic solutions, where “one size fits all,” will require extensive clinician collaborations and consensus. Detailed feedback from the various methodologies that exemplify these points is presented in Textbox 3.

**Impact of the tool on patient care.**

 

**Positive impact on patient care**

- Having a problem list improves patient care and safety
  “In general surgery we need to know about a lot of other specialties. If these were summarised in a problem list this would really help” [upper gastrointestinal surgeon]
  “From a clinical point of view, it ought to be a good thing certainly because just looking at what is in ICD 10 which is what we were using, there is an awful lot in there that really is not clinical classification suitable for individual patients whereas in SNOMED that should be, they should all be suitable for individual patients for at least the disease and the implications that we are using should be. So yes, I think that it is probably worthwhile” [programmer]
  “Yes useful if its filled out for example, steroids – if these are missed but are on the problem list this would be very useful” [upper gastrointestinal surgeon]
  “If you get wrong things in the record, like ‘heart attack’ this may prevent chemotherapy in the future” [consultant oncologist]
- Correct coding has implications for safety rules
  “Once this replaces paper notes as a single instance of truth, it will be very valuable and can be used to drive decision support and indication based prescribing or decision support” [doctor]
- Improvement in communication
  “I think having the problem list is very helpful, but I think a clerking page on prescribing information and communication system for the admission would be very helpful especially with patients who have been in for a while” [user]
  “For people in highly specialised specialities they don’t cross over into other peoples’ fields. In general surgery we need to know about a lot of other specialities. If these were summarised in a problem list this would really help” [upper gastrointestinal surgeon]
  “For ED/pre-assessment - this is where it’s so important to document this” [neurologist]
  “If you saw a new patient with 30-40 letters you would be missing very important information that you would really want to know” [upper gastrointestinal surgeon]
  “I think it’s important because when people go away and come back lots of important information is there” [HIV consultant]
  “The problem is sometimes not too much information but too little” [hand surgeon]

 

**Negative impact on patient care**

- Lists may get too long
  “Terms are so detailed that a patient could end up with several codes for the same diagnosis, and this will make the problem list overly complicated and the letters cluttered” [consultant geriatrician]
  “It’s lovely to have this list in the medical record but I’m not sure a 30 year list is all relevant and can lead to confusion, failure to triage and fatigue” [consultant nephrologist]
- Human nuance may be lost
  “For me in cardiology I do detailed problem lists. There is not sufficient detail in this tool” [cardiologist]
  “It’s difficult you can’t replicate the problem list [that a human created] at the top of the clinical letter without there being too much information” [consultant nephrologist]
  “Yes we need more detail on the lists we need the notes ‘this patient requires steroids’ – is this part of SNOMED?” [upper gastrointestinal surgeon]
  “I don’t use this at all I only use my letters. I only summarise for my specialty” [liver medicine]

### Use in Research or Audit

The principal concern regarding the use of codes for research was the overlapping of codes and lack of granularity. However, users also noted that the tool was already being used in audits*.* Many clinicians have recognized the potential for research and expected that digital development could contribute extensively.

### Use in Data Sharing

In the questionnaire, users generally thought that the tool would improve data exchange (Figure 3). It was generally agreed that more standard and detailed coding made data sharing easier and that this was desirable for patient care. Users were interested in sharing the data with general practitioners and other hospitals. Clinicians were also interested in the idea that data could be pulled into the hospital system for acute and outpatient care*.* User feedback is presented in Textbox 4. Of note is that users remained concerned about the number of codes available, although there is some recognition that these can be mapped to each other or grouped for the purposes of research and clinical care.

### Standards and Payment

Data sharing was also discussed in relation to payments and uploads to national registries. Programmers were the only group that discussed the NHS standards (Textbox 4)*.*

**Use in research, audit, data sharing and payment.**

**The tool is useful in research**

- “The only difficultly I find with the patient group I see is that there tend to be multiple overlapping codes which could be used and unless easy consensus is reached across the organisation you may get lots of codes of one condition. We used to be able to see the 'code' behind the 'description'. This would still be useful, particularly for future informatics searches based on code” [doctor]
- “Assuming it is used correctly it will be helpful (more for research than for clinical care) but it can be tricky to know what to code and how to code - there are often a lot of similar diagnoses on SNOMED which can make it confusing” [user]
- “I used to use this list in order to code patients with a view to doing audit on out patients - however when we have requested the list of patients with a certain diagnosis for audit purposes we are told that we can only search for in patients this way. As a result people have stopped coding outpatient episodes as they can no longer see the relevance. It would be good to be able to generate a list of certain outpatient diagnoses on request so that we can improve patient care” [user]
- “More granularity for research is likely to be needed but with less overlap” [user]
- “Used by NIV [non-invasive ventilation] physio team to record patient as receiving acute NIV data. This should allow data to be collated for the COPD [chronic obstructive pulmonary disease] national audit. I would like to know if it can be used locally to pull data for NIV service audit at QEHB [Queen Elizabeth Hospital Birmingham]” [user]
- “If the information was structured this would be a valuable source of data for any future admissions, audit and research” [acute medicine consultant]
- “The more coded information we have the more useful it will be for the future – information in the system means computers will be able to pull this and write queries, your own brain can’t do this when there is too much information” [consultant nephrologist]
- “In the old days there was no information, computing has revolutionised this but there is now irrelevant data overload” [HIV consultant]

**Data sharing**

- In general
  “Well certainly in terms of sharing, the codes that we are using should have, it is difficult to say agreed semantics but there should be less disagreement over what the semantics of them are. Certainly within the use of local codes that were being used maybe in terms of ICD10 codes, I can think of some ICD10 codes that, depending on whether you interpret the description of the code as being about a single patient or about a patient population which are completely different things because of the use of the word and where it kind of gets turned around when it is a bucket to really mean for a single patient” [programmer]
  “It is something which seems to be highly detailed so presumably is going to make it easier for sharing data between systems which is, I guess it has to be an aim in the long run for most organisations” [programmer]
  “So we must facilitate data sharing and this improves patient care doesn’t it?” [hands surgeon]
- With general practitioners
  “Discharge codes should go into letters and be sent to the GP” [fixed point data]
  “Also by definition it would be nice if that were put on the discharge letter (not that I usually see what is actually on them), but I guess that by “finishing” the code, it isn’t put anywhere?” [consultant in hand and plastic surgery]
  “GP Systems are really clunky, this needs to be transcribed, if this was intelligent it would help GP colleagues a lot” [HIV consultant]
- With other health care providers
  “So I think I understand how this feeds into data sharing. Improving clinical care – when I go to the Women’s Hospital I am completely blind, if this could join things together that would be great” [consultant nephrologist]
  “So the Ambulance Service have very little understanding about what has gone on with the patient, and end of life. We don’t know the post mortem data” [hands surgeon]
- To pull data into the organization
  “Prescribing information and communication system needs to be able to pull things in from other systems” [plastics and hands surgeon]
  “We need the clerking in emergency department” [vascular surgeon]
  “On the post take ward round we are checking letters, if it was there it would be much quicker” [upper gastrointestinal surgeon]
  “Confused elderly patient, this is important when you are trying to get GP information – it will be fantastic” [ICU consultant]
  “There are lots of patients who have had other things in other hospitals, but no proper data sharing exists” [consultant cardiologist]

**Standards and payment**

- “Plus it is mandated by the National Health Service” [programmer]
- “Well, yes it is something that has been mandated” [programmer]
- “If you do things incorrectly the clinical commissioning group will take money off you” [upper gastrointestinal surgeon]
- “We have been caught out by poor coding” [ICU consultant]
- “In Transcatheter Aortic Valve Implantation the list is strange for payment, on prescribing information and communication system we list these in the right order so that the coders get it right – this is very difficult and specific to do – it’s all manual. It’s now a game – there are points” [consultant cardiologist]
- “One of the issues is that codes may be hidden in notes and you might not get your money” [upper gastrointestinal surgeon]

### The Importance of Various Themes to Users

To understand which aspects of the tool were most important to users, feedback from the focus groups, interviews, emails, and free comment text in the questionnaires were marked to understand where comments or conversation had been based on particular themes, and analyzed for time spent (Figure 1). In the focus groups and interviews, this was measured as the actual time spent in discussing an item; in the questionnaire, and email feedback, this was the length of free text comments written by respondents.

Overall, users were most concerned about the ease of use of the tool and its direct use. They were then equally concerned with patient care, data sharing, and further development. All users talked about all the themes except for programmers who, as expected, did not talk about usefulness, as they were not users. Questionnaire comments focused on how easy it was to use the tool, with some focus on usefulness and patient care.

Interestingly, the 2 focus groups spent time on different themes, despite having been shown the same facilitation tools. One group discussed data sharing extensively and the other future developments. Neither focus group discussed patient care in depth, which may be because of the seniority of the doctors in these groups, for whom the importance of good patient care is already assumed to be high.

### Validity and Reliability of Data

Data were triangulated from fixed-point data sets, surveys, interviews, and focus groups with the data collected on actual use statistics. Survey data were collected until saturation or redundancy was reached. To reduce nonresponder bias, the survey was constructed carefully using proven techniques to ensure experience, opinion, and emotional response without deploying leading questions or resulting in dead-end answers [16]. Reminders were deployed to increase response rates, and the survey was deliberately short so that it was easy to complete. The survey was digital to ensure the ease of returning completed responses. The principal investigator has a relationship with many clinicians across the organization, and this was exploited to increase response rates. Member checks ensured internal validity by soliciting feedback on preliminary findings from participants to check the correct interpretation [23].

## Discussion

### Principal Findings

This study demonstrates the successful conversion of a hospital electronic record to SNOMED-CT with high clinician acceptability, which forms the basis of data sharing, innovative use of data, and availability of coded clinical information for research.

SNOMED-CT as an ontology has been developed over several decades [24] and represents a detailed and multifaceted database that can be used to accurately describe patients’ conditions, medical and surgical history, and current problems. The ontology is divided into several levels, allowing both specific detail and broader general categories, and these are linked together in a web structure [7].

There are several coding structures currently in use in the English NHS, including the *ICD-10* and Office of Population Censuses and Surveys Version 4, which are the current methods of payment for hospitals [13,25]. In general, these methods do not provide the depth or detail required to describe patient conditions. This lack of detail leads clinicians to look for better ways to encode clinical information [26-28].

### Data Exchange

Clinicians and patients increasingly demand safe exchange of clinical data to optimize clinical care [29]. This can be leveraged in secondary data collection for research to safeguard future population health [30]. The interoperability of health systems, and in particular, moving data between systems, has been discussed extensively but has not been realized to date [4]. One of the major hurdles is nonstandard data collection [31]. It is difficult to transfer data where mapping from one coding system to another is needed, especially when the mapping is from one generalized diagnosis to many more detailed ones. In an ideal situation, the primary data sources would collect data in a single standard coding structure, facilitating easy data exchange [14]. Clinicians in our study recognized the need for data exchange and welcomed a tool that actively facilitates sharing.

The national strategy in England is clearly set out by NHS Digital with aspirations for all aspects of the English NHS to use SNOMED-CT from 2020. Conversion is under way in primary care and secondary care, acute care, mental health, community systems, dentistry and other systems used in direct patient care must use SNOMED-CT as the clinical terminology, before April 1, 2020 [7]. The conversion of commercial systems has been slow [32], but continues to increase.

### Previous Transitions

There are no published examples of SNOMED-CT conversion in a complex hospital EHR that supports CDS, but there are several case studies of smaller successful and unsuccessful implementations of SNOMED-CT in EHRs. Lee et al [33] studied 13 implementations, 5 of which were hospital-wide, and 4 of these involved diagnostic lists. Clinicians found that SNOMED-CT did not always include all diagnoses that they required. In a small Portuguese study [34], user acceptance was high, and the adoption of SNOMED-CT enabled system interoperability. More recently, in Wales, the successful development of a neurology database using SNOMED-CT has influenced direct patient care, allowing accurate web-based reporting and informing clinical research [35].

Currently, there is very little published literature and there are no peer-reviewed papers describing the impact of primary or whole system secondary care transition to SNOMED-CT in England. Many of these implementations also reported benefits not directly related to SNOMED-CT, but rather to transition from paper to digital recording [33]. The principal trends from previous implementations were reflected in our study—the hierarchy was challenging to use, some concepts were ambiguous, and there was some syntactic inconsistency. The combining of multiple terms used in SNOMED-CT was also challenging and consequently created difficulties in data retrieval [33-35]. Success trends could also be found; implementations were successful as the UI was simple and immediate value was demonstrated to clinicians. Synonyms allowed clinicians to easily find terms and reuse the data.

### Interface

Clinicians not only require a coding system that encompasses all the diagnoses and procedures that affect patients; they also want a seamless UI for data entry [33]. By providing a more complex and detailed coding structure, an unintended consequence may be that the data entry application is so complicated that clinicians disengage, resulting in a paradoxical decrease in acquired data [36,37]. The SNOMED-CT database is complex and needs to be presented in a way that makes sense to clinicians [38,39]. The interface must be intuitive and not overwhelming, and the search engine must return specific terms in a helpful order [40]. Data exchange must be considered at inception [4], and mapping back to other coding structures must be possible, for example, to national data sets for reporting [41] and to the *ICD-10* for payment [42]. Of note, where enriched data are collected by clinicians, mapping back to the *ICD-10* for payment can be effective in increasing hospital income [43]. Reflecting these points, we found in our study that clinicians accepted the use of SNOMED-CT, but care was needed in the presentation and utility of this vast database in clinical EHR. In general, clinicians report success in using the tool, finding the depth of the coding useful and applicable [33]. Clinicians, particularly doctors, are in general interested primarily in providing excellent patient care, and drivers for successful projects are therefore efficient patient safety [26,44] and research for population health [45].

The organization has a long history of user-led design, and the strength of the EHR is that it is “designed for clinicians by clinicians.” Increasingly successful digital health systems are reported to have a human-based design at their core [4,46-48]. Bringing developers and users together is reported to increase usability and value [49,50], forming the basis of user-based co-design [51].

### Embedded in Complex EHR With CDS

In addition to the literature on SNOMED-CT and its implementation, the design of the EHR is crucial to the success of the project. Independent of the coding structure behind it, the EHR must be highly usable to engage clinicians, and must be intuitive and helpful or risks disengagement [3]. Data sharing for direct health care, or for population research to safeguard future generations, is only possible if data are entered into the record in the first place. Therefore, interface design is crucial [40,46], more so in implementations involving extremely complex coding systems such as SNOMED-CT. In addition to design, standards that are universally adopted (or enforced) are required to allow data sharing and interoperability of systems to work [5,8,52]. Sophisticated CDS or artificial intelligence algorithms cannot be leveraged if clinical records are devoid of data.

Beyond the transition of EHR to SNOMED-CT, there is limited literature on its subsequent use in CDS and artificial intelligence. Previous studies in this area have briefly discussed the use of SNOMED-CT in CDS [53,54], but have not explored the complex mapping that is needed to link existing rules to multiple codes as a result of the one-to-many mapping. SNOMED-CT has the potential to greatly enrich medical records [34] and therefore enhance the safety and quality of care via CDS [53]. The detailed diagnostic codes in SNOMED-CT may also allow the development of more precise CDS in the future. The extensive work needed to map all the codes in an existing system was completed, although it took time.

In addition, the transition of complex EHR to SNOMED-CT must consider some unique problems. SNOMED-CT is compositional, negating the need for a large number of specific terms. Therefore “left pneumothorax as a complication of a chest drain” does not exist in SNOMED-CT; terms must be combined to reach concepts that are applicable to patients. Compositional systems allow greater reuse of data without the need for human intervention for the interpretation of the categories [55]. The challenge is to allow clinicians to choose from the richness of the database, including combining of concepts, while safeguarding the CDS rules so that any concept related to the conditions in the rule is fired by all of the relevant SNOMED-CT codes that clinicians choose.

### Health Ecosystem

This project took place in a large secondary and tertiary care hospital ecosystem consisting of 4 hospitals and community services. This ecosystem has nascent links to primary care records via a project that does not yet exchange data but uses a “look up and leave” data sharing platform [56]. Beyond the institution itself, the EHR has been acquired by 2 other hospitals in the West Midlands [57,58]. Similar ecosystems in the United Kingdom are now increasingly implementing EHR [59,60], and in North America, many have already done so [61]. There are limited examples of acute care ecosystems that implement a change in coding, but a few exist [34,62]. None of these studies, however, achieved recoding in a complex EHR that supported complex CDS rules, and none of these examples reported on data exchange after the coding change.

Of note, although this study is restricted to the acute hospital setting, the whole of the NHS is influenced by projects of this kind. By converting coding central to one of the largest acute trusts in the country whose influence through its software extends beyond the institution itself, this project contributes to the facilitation of national data exchange.

### Limitations and Delimitations

The limitations of this study are largely related to the time-limited nature of the study; thus, a pragmatic approach had to be taken for the number of respondents for the survey and the number of focus groups that could be undertaken. The study protocols aimed to continue the survey until saturation was reached and to reduce bias from nonresponders. However, this was not entirely possible, and the study therefore needed to focus on deep analysis of smaller data sets rather than unrealistic expectations of analyzing extensive data sets.

Limitations beyond the control of the investigator were the truthfulness of the responders, the attendance of participants to invited groups, and consent for research inclusion.

The research was only able to study the response to the new tool for the first 4 months after its launch. This may not have been representative, as novel software may be used extensively initially with a reduction in use over time, or conversely, may increase as more clinicians become committed to data sharing. Pre-existing data sets were brief notes, capturing requirements for software build and modification. Although imperfect, these were valuable resources for comparing the attitudes and requirements of both stakeholders before and after the launch of the tool. Finally, the study could not study the repetitive loops of development, and it is likely that iterative changes to the tool are needed.

### Lessons Learned

The following lessons were learned during the development and implementation process:

1. Clinicians will accept the task of adding SNOMED-CT–coded diagnoses into an electronic record provided the UI is intuitive, but they will disengage if the process is too complex.
2. The underlying EHR must be highly usable to engage clinicians.
3. Bringing developers and users together increases usability and value by engaging in user-based co-design.
4. Clinicians are driven by patient safety, data exchange across the health system, and research for population health.
5. Sophisticated and CDS or artificial intelligence algorithms cannot be leveraged without this data collection.

### Conclusions

This study demonstrates the successful conversion of a hospital electronic record to the updated standardized coding of diagnoses, with high clinician acceptability. The project began with clinician demand, followed by the building of a clinically led tool and extensive examination of its success and requirement for iteration. Starting in one institution, this has implications for the entire NHS. This tool is important because it supports national aspirations for enhancing patient care through CDS and allows for data sharing, innovation, and research. The project is a blueprint for capturing the data that form the bedrock for driving artificial intelligence and full digitalization of health care, and it is therefore a benchmark for transformation and innovation, influencing the aspiration to safeguard health for future generations.

## Appendix

Multimedia Appendix 1 Fixed documents and excerpts from specification documents (user stories).

Multimedia Appendix 2 Survey of clinicians regarding acceptance of the problem list tool in the prescribing information and communication system.

Multimedia Appendix 3 Interview questions for developers working on the project.

## Abbreviations

AHP: Allied Health Professionals
CDS: clinical decision support
EHR: electronic health record
ICD-10: International Classification of Diseases, Tenth Revision
NHS: National Health Service
PICS: Prescribing Information and Communication System
SNOMED-CT: Systematized Nomenclature of Medicine–Clinical Terms
UHB: University Hospitals Birmingham
UI: user interface
