# Supplementary material for: Introduction of Systematized Nomenclature of Medicine–Clinical Terms Coding Into an Electronic Health Record and Evaluation of its Impact: Qualitative and Quantitative Study
Source: JMIR Med Inform. 2021 Nov 23;9(11):e29532. doi: 10.2196/29532 (PMC8663536; doi:10.2196/29532)
Supplement: Multimedia Appendix 1 [file medinform_v9i11e29532_app1.docx]

## Appendix 1: Fixed documents, excerpts from specification documents (User Stories)

MosCow prioritisation: Must have, Should have, Could have, Won’t have (but would like).

| **As a/an** | **I want** | **so that** | **MoSCoW** |
| --- | --- | --- | --- |
| **Doctor/Nurse/Therapist** | QuickList Patient Classifications to be SNOMED CT codes | Classifications are all SNOMED CT codes & descriptions | Must Have |
| **Doctor/Nurse/Therapist** | Contraindications rules mapping to work with SNOMED CT codes | PICS rules trigger on SNOMED codes | Must Have |
| **Doctor/Nurse/Therapist** | All MLM’s rules to work with SNOMED CT codes | PICS rules trigger on SNOMED codes | Must Have |
| **Doctor/Nurse/Therapist** | To change PICS so that we have a single tab which combines comorbidities and classifications (local and ICD10 codes) – this tab should be called “Health Conditions”. | We have a single tab which combines comorbidities and classifications | Must Have |
| **Doctor/Nurse/Therapist** | I want to select from SNOMED CT codes wherever possible.  *Note – The local list should reduce significantly, it has grown due to lack of options in ICD-10. However some local codes will remain to cover miscellaneous items that cannot map to SNOMED.* | We use a more comprehensive set of classification codes. | Must Have |
| **Doctor/Nurse/Therapist** | To be able to use a SNOMED CT search facility from within PICS. | I can select the most appropriate SNOMED CT code for the patient diagnosis or procedure | Must Have |
| **Doctor** | On first entry to a patient record I want to see historical ICD-10 codes displayed (greyed out) and see a suggested (not mandatory) matching SNOMED code that could be selected to generate a classification.  If the new suggested SNOMED classification is selected it will appear to replace the original ICD-10 code and assume the original (ICD-10) start date. It will not kick-off new PICS rules, and the old ICD-10 code will be removed from the patient’s Health Conditions list.  For ones that do not map, options presented to the clinician in a drop down list so that they can select one. Offering a sensible SNOMED alternative to make it as easy as possible for them to resolve.  To stop ICD-10 codes from being able to be selected for new cases. | All codes selected post Go-Live are SNOMED CT. | Must Have |
| **Doctor/Nurse/Therapist** | A data cleanse of SNOMED data to remove Veterinary elements, Drugs, Devices, etc | They do not display in PICS SNOMED search results | Must Have |
| **Doctor/Nurse/Therapist** | To change the search results pane to show SNOMED + Local Classifications side by side | They are easier to view | Must Have |
| **Doctor** | All Procedure codes to be SNOMED CT  Need to include date that procedure was conducted. Also Procedure outcome box needs to be filled in if we are invoking SNOMED codes*.* | We standardise on SNOMED CT coding for procedures | Must Have |
| **Doctor** | 1. Discharge codes to go on the Health Conditions list as classifications. 2. SNOMED descriptions to go on the discharge letter to the GP | Discharge diagnosis is improved | Must Have |
| **Doctor/Nurse/Therapist** | The implied description & codes should not be used in letters.  I do not want implied codes displayed on the active patient list. | Letters don’t contain implied information that clinician has not approved  Implied classifications are only displayed if used by a rule. | Must Have |
| **Doctor/Nurse/Therapist** | The current column on the active patient list collapsed and renamed to ‘Health Conditions’ and moved to the left next to the Doctor’s handover notes. It should display the first line, and then display the remainder if you hover over it. | Improved information display | Must Have |
| **PICS Developer** | A ‘middleman routine’ to support contraindications & MLM rules triggering for original codes and new corresponding SNOMED codes.  Must still work for ICD-10 codes too. | Rules trigger on SNOMED codes, and also trigger on the old ICD-10 codes too. | Must Have |
| **Doctor/Nurse** | The active patient list not to display the code in the classification column, just the description.  The messages & tasks column to be expanded so there is room for longer descriptions of tasks. |  | Must Have |
| **Doctor/Nurse** | The Health Conditions display needs to be in sections like, ‘previous operations’, ‘previous medical conditions’, etc, with the ability to be able to sort these by date if desired. | So that old procedures & classifications are easily identified | Must Have |
| **Doctor/Nurse** | Refreshed admission reasons by Directorate | So that we have a tidy sensible list per specialty | Must Have |
| **Doctor/Nurse** | Admission codes that do not have a SNOMED equivalent e.g 5% - 10% Burns are not used regularly. Feedback from Burns & Plastics is that the percentage  range for burns is not useful at all on the admissions screen, as they we have better definition of percentage under (clinical). Therefore the standard SNOMED codes for Burns should suffice, and the tick box percentage burns can be removed. | So that we have a tidy sensible list per specialty | Must Have |
| **PICS Developer** | To migrate all legacy procedures via a script, the script will need to pull these out, map to SNOMED, convert to a classification and populate the Health Conditions list as a ‘previous procedure’.  It is envisaged the bulk of the legacy procedures can be imported well ahead of Go-Live, with only a small top-up needed at Go-Live time. | So that legacy procedures appear on the Health Conditions list. | Must Have |
| **Doctor** | Health Conditions list that is sortable by date and also alphabetically | To make important items more visible | Must Have |
| Surgeon | Laterality  To be able to select laterality at Admission time e.g for a fractured neck of femur….be able to select Left or Right. SNOMED does not contain different codes for left and right so we will need a developed solution such as an Admissions tick box for left & right? or selectable radio buttons. | It is clear which side of the body is being referred to | Should have |
