# Supplementary material for: Introduction of Systematized Nomenclature of Medicine–Clinical Terms Coding Into an Electronic Health Record and Evaluation of its Impact: Qualitative and Quantitative Study
Source: JMIR Med Inform. 2021 Nov 23;9(11):e29532. doi: 10.2196/29532 (PMC8663536; doi:10.2196/29532)
Supplement: Multimedia Appendix 2 [file medinform_v9i11e29532_app2.docx]

## Appendix 2: Survey of Clinicians regarding acceptance of Problem List tool in PICS

| 1. Have you used the new ‘problem list’ in PICS? 2. Is it easy to find in PICS? 3. Does the SNOMED-CT search tool allow you to find patients diagnoses or problems easily? 4. Is the problem list displayed in the right places in PICS? 5. If ‘no’ where should this be displayed? (free comment) 6. How likely do you think this will be to improve patient care? 7. How likely do you think this will be to improve data exchange and research? 8. Have you got any other feedback? |
| --- |
