# Supplementary material for: Introduction of Systematized Nomenclature of Medicine–Clinical Terms Coding Into an Electronic Health Record and Evaluation of its Impact: Qualitative and Quantitative Study
Source: JMIR Med Inform. 2021 Nov 23;9(11):e29532. doi: 10.2196/29532 (PMC8663536; doi:10.2196/29532)
Supplement: Multimedia Appendix 3 [file medinform_v9i11e29532_app3.docx]

## Appendix 3: Interview questions for developers working on the project

| 1. What is your role in the organization, and how long have you worked here? 2. What is your role in the project? How many hours a week are you/did you work on this? 3. What is your opinion on the success of the project? 4. What has been done well? 5. What are you unhappy about? 6. Why is the project important? 7. What are the most important aspects of the project? 8. What is your view on the users experience using our new tool? |
| --- |
